# Supplementary material for: Investigating the Molecular Mechanisms of Resveratrol in Treating Cardiometabolic Multimorbidity: A Network Pharmacology and Bioinformatics Approach with Molecular Docking Validation
Source: Nutrients. 2024 Jul 31;16(15):2488. doi: 10.3390/nu16152488 (PMC11314475; doi:10.3390/nu16152488)
Supplement: Supplementary file 1 [file nutrients-16-02488-s001.zip › Supplementary Figures S1 and S2.pdf]

Supplementary Figures

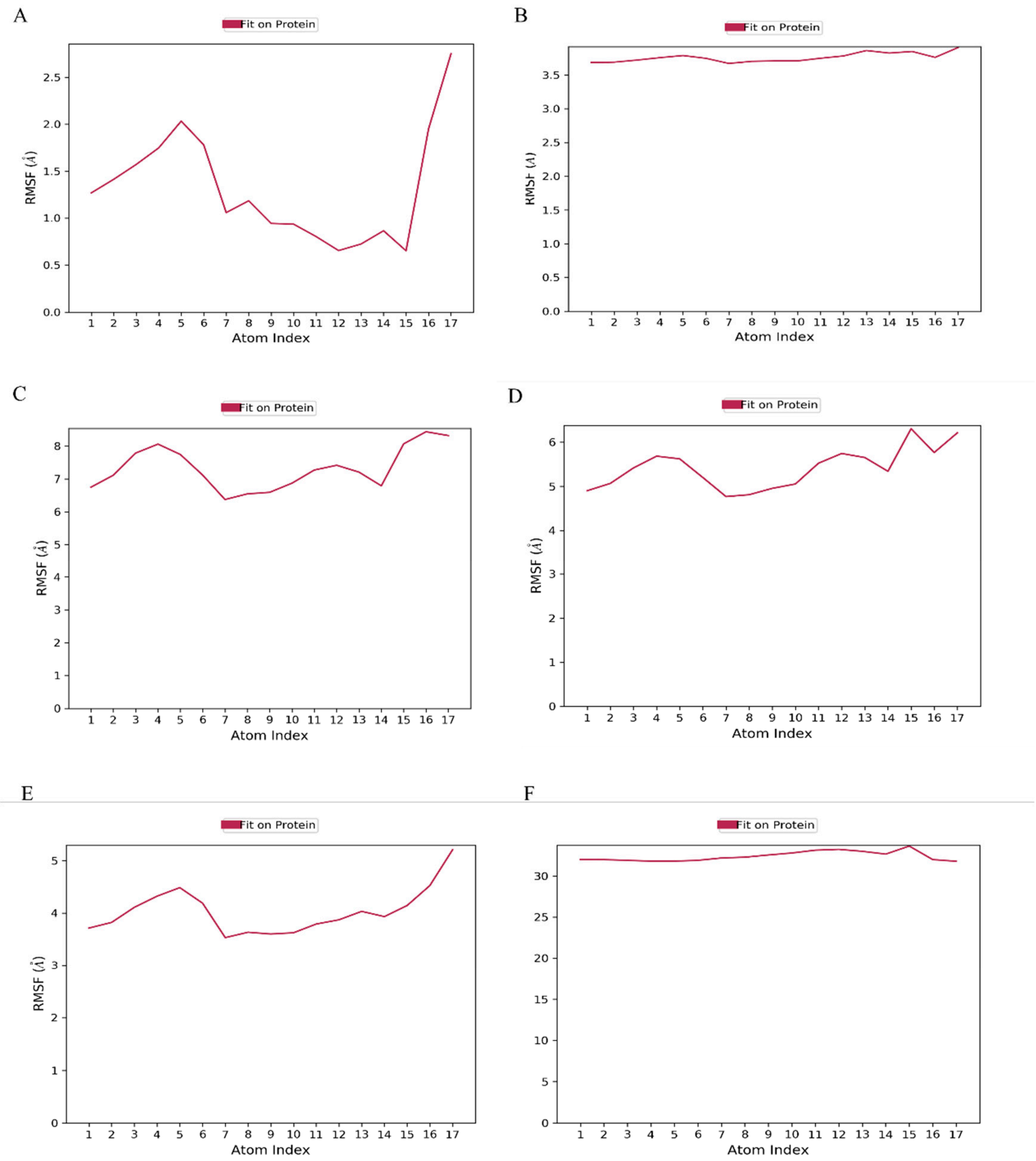

Figure S1. RMSF of ligands(A) MAKP3 (B) EGFR (C) FGFR1 (D) FGF2(E) STAT5 (F) STAT3

A

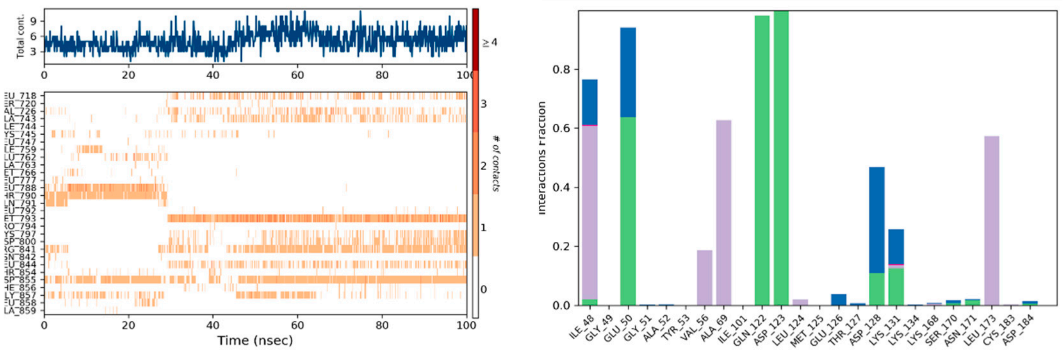
Total cont.
Time (nsec)
# of contacts
Interactions Fraction
ILE_48 GLY_49 GLU_50 GLY_51 ALA_52 TYR_53 VAL_56 ALA_69 ILE_101 GLN_122 ASP_123 LEU_124 MET_125 GLU_126 THR_127 ASP_128 LYS_131 LYS_134 LYS_168 SER_170 ASN_171 LEU_173 CYS_183 ASP_184

B

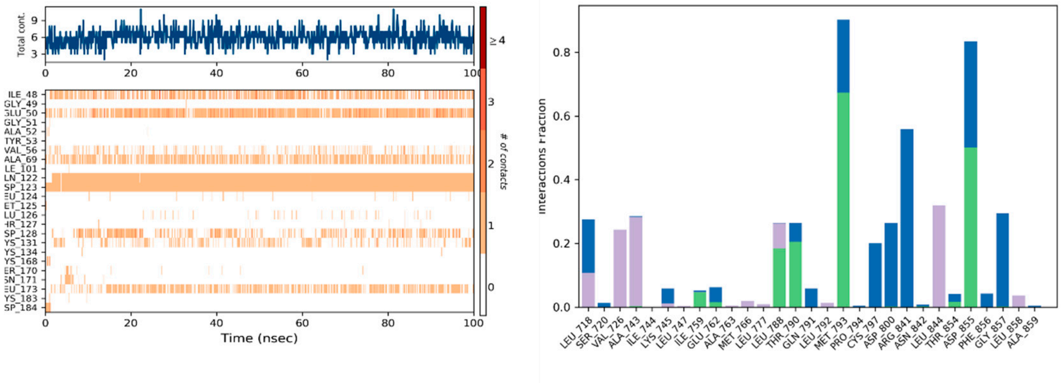
Total cont.
Time (nsec)
# of contacts
Interactions Fraction
LEU_718 SER_720 VAL_726 ALA_743 ILE_744 LYS_745 LEU_747 ILE_759 GLU_762 ALA_763 MET_766 LEU_777 LEU_788 THR_790 GLN_791 LEU_792 MET_793 PRO_794 CYS_797 ASP_800 ARG_841 ASN_842 LEU_844 THR_854 ASP_855 PHE_856 GLY_857 LEU_858 ALA_859

C

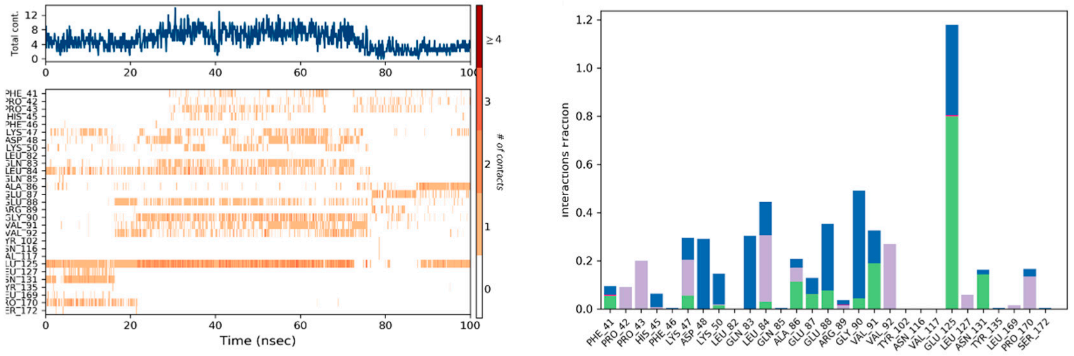
Total cont.
Time (nsec)
# of contacts
Interactions Fraction
PHE_41 PRO_42 PRO_43 HIS_45 PHE_46 LYS_47 ASP_48 LYS_50 LEU_82 GLN_83 LEU_84 GLN_85 ALA_86 GLU_87 GLU_88 ARG_89 GLY_90 VAL_91 VAL_92 TYR_102 ASN_116 VAL_117 GLU_125 LEU_127 ASN_131 TYR_135 LEU_169 PRO_170 SER_172

D

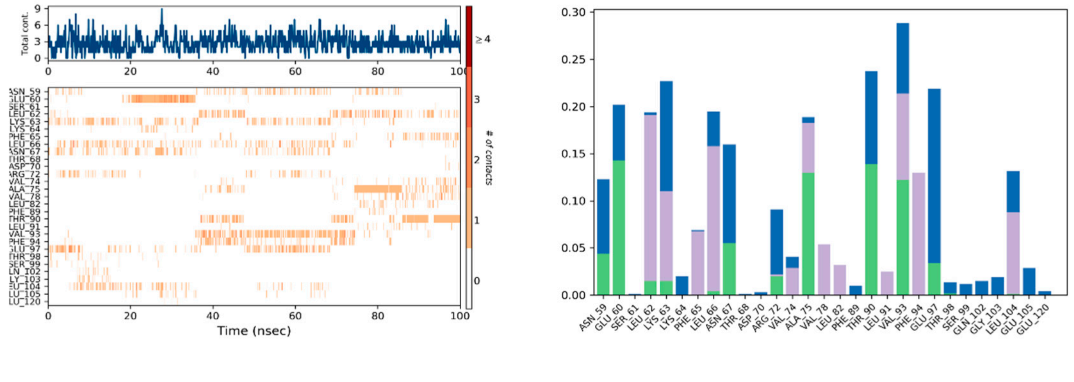
Total cont.
Time (nsec)
# of contacts
ASN_59 GLU_60 SER_61 LEU_62 LYS_63 LYS_64 PHE_65 LEU_66 ASN_67 THR_68 ASP_70 ARG_72 VAL_74 ALA_75 VAL_78 LEU_82 PHE_89 THR_90 LEU_91 VAL_93 PHE_94 GLU_97 THR_98 SER_99 GLN_102 GLY_103 LEU_104 GLU_105 GLU_120

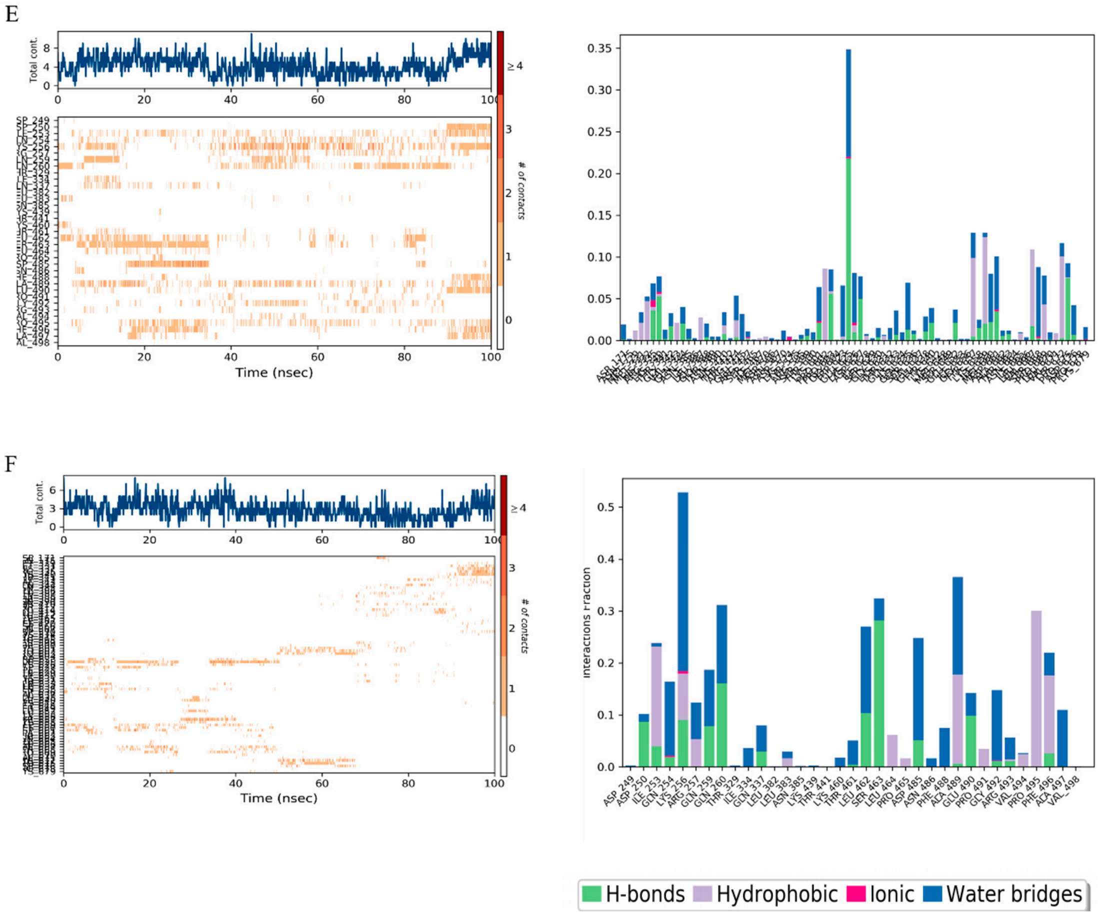

Figure S2. PL-Contacts (A) MAKP3 (B) EGFR (C) FGFR1 (D) FGF2(E) STAT5 (F) STAT3
