# Supplementary material for: Investigating the Molecular Mechanisms of Resveratrol in Treating Cardiometabolic Multimorbidity: A Network Pharmacology and Bioinformatics Approach with Molecular Docking Validation
Source: Nutrients. 2024 Jul 31;16(15):2488. doi: 10.3390/nu16152488 (PMC11314475; doi:10.3390/nu16152488)
Supplement: Supplementary file 1 [file nutrients-16-02488-s001.zip › Supplementary Table S16.pdf]

Supplementary Table S16
Pathway enrichment results for MCODE analysis

Color	MCODE	Go	Description	Log10(P)
	MCODE1	hsa04080	Neuroactive ligand-receptor interaction	-23.2
	MCODE1	hsa04630	JAK-STAT signaling pathway	-8.6
	MCODE1	hsa04060	Cytokine-cytokine receptor interaction	-5.6
	MCODE2	hsa04935	Growth hormone synthesis, secretion and action	-12.4
	MCODE2	hsa04630	JAK-STAT signaling pathway	-9.4
	MCODE2	hsa04917	Prolactin signaling pathway	-9.3
	MCODE3	hsa05143	African trypanosomiasis	-6.0
	MCODE3	hsa05144	Malaria	-5.6
	MCODE3	hsa05166	Human T-cell leukemia virus 1 infection	-5.3
	MCODE4	hsa05218	Melanoma	-32.6
	MCODE4	hsa04015	Rap1 signaling pathway	-29.2
	MCODE4	hsa04014	Ras signaling pathway	-28.5
	MCODE5	hsa03420	Nucleotide excision repair	-5.7
	MCODE6	hsa05010	Alzheimer disease	-5.3
	MCODE6	hsa04114	Oocyte meiosis	-5.0
	MCODE6	hsa04022	cGMP-PKG signaling pathway	-4.7
	MCODE7	hsa05145	Toxoplasmosis	-10.1
	MCODE7	hsa04935	Growth hormone synthesis, secretion andaction	-10.0
	MCODE7	hsa04380	Osteoclast differentiation	-9.7

	MCODE9	hsa00480	Glutathione metabolism	-13.7
	MCODE10	hsa05410	Hypertrophic cardiomyopathy	-6.5
	MCODE10	hsa05414	Dilated cardiomyopathy	-6.4
	MCODE10	hsa04820	Cytoskeleton in muscle cells	-5.4
	MCODE11	hsa04060	Cytokine-cytokine receptor interaction	-8.0
	MCODE11	hsa04350	TGF-beta signaling pathway	-6.8
	MCODE11	hsa04390	Hippo signaling pathway	-6.3
	MCODE12	hsa04610	Complement and coagulation cascades	-7.1
